# Examining the correlation between diabetes and odontogenic infection: A nationwide, retrospective, matched-cohort study in Taiwan

**DOI:** 10.1371/journal.pone.0178941

**Published:** 2017-06-08

**Authors:** Hui-Hsin Ko, Wu-Chien Chien, Yen-Hung Lin, Chi-Hsiang Chung, Shih-Jung Cheng

**Affiliations:** 1 Graduate Institute of Clinical Dentistry, School of Dentistry, National Taiwan University, Taipei, Taiwan; 2 Department of Dentistry, National Taiwan University Hospital, College of Medicine, Taipei, Taiwan; 3 Department of Dentistry, National Taiwan University Hospital, Hsin-Chu Branch, Taiwan; 4 School of Public Health, National Defense Medical Center, Taipei, Taiwan; 5 Department of Oral and Maxillofacial Surgery, Linkou Chang Gung Memorial Hospital; 6 School of Dentistry, National Taiwan University, Taipei, Taiwan; 7 Graduate Institute of Oral Biology, School of Dentistry, National Taiwan University, Taipei, Taiwan; Kagoshima University Graduate School of Medical and Dental Sciences, JAPAN

## Abstract

More than 90% of head and neck infections are caused by pathological changes originating in the teeth. When odontogenic infections are not properly treated, infections may spread to distant spaces and cause more serious infections in fascial spaces, ultimately leading to deep neck infections. Clinical experience has indicated that patients with diabetes mellitus (DM) may be more susceptible to facial cellulitis and deep neck infections caused by odontogenic infections. This study used the Taiwan National Health Insurance Database (NHIRD) to analyze and examine the correlation between DM and odontogenic infections in patients. To this end, this study analyzed 1 million NHIRD individual datasets from 2005, of which 964,182 individuals had medical treatment records. The insurance database also recorded related factors such as age, sex, duration of hospital stays, season, and whether patients were low income. We also analyzed the correlation between urbanization and the studied diseases. The results indicated that the correlation between facial cellulitis and DM patients was confirmed; facial cellulitis was most likely to occur 2 years after the initial DM diagnosis, with a risk occurrence 1.409 times greater than that of the control group. Facial cellulitis is more likely to occur in patients originating from poorer socioeconomic backgrounds, and female DM patients are more likely to experience this condition. These conclusions may facilitate the establishment of clinical guidelines for preventative education and treatment. Oral prevention and health education for high-risk patients, as well as early-stage surgical intervention and antibiotic usage in early-stage odontogenic infections, can prevent disease progression, improve patient recovery rates, and reduce the use and waste of medical resources.

## Introduction

Odontogenic infections are caused by dental caries, periodontal disease, radicular cysts, and other oral diseases [1–3]. Minor cases of odontogenic infection can lead to the formation of abscesses or facial cellulitis, while severe cases may result in often fatal deep neck infections. More than 90% of head and neck infections are caused by dental pathological changes [4]. When odontogenic infections are not properly treated, they may spread to distant locations, causing more serious infections in fascial spaces and ultimately leading to deep neck infections. As a result, when patients present with pain and swelling in the head and neck accompanied by difficulties in opening the mouth or swallowing, the possibility of odontogenic tissue infection must first be investigated. Approximately 40% of odontogenic infections lead to deep neck infections [1,5–7], causing systemic sepsis or upper respiratory distress that threatens the lives of patients. Although the use of antibiotics is widespread, the desired effects may not be attained in deep tissue infections.

In the field of dental treatment, when patients have underlying diseases such as diabetes mellitus (DM) or cancer, compromised immune systems may lead to the opportunistic progression of seemingly minor infections. The possibility of oral abscesses progressing into deep neck infections increases with the rate of progression. Not only does this phenomenon increase the difficulty and complexity of treatment, it also exposes patients to the risk of death and side effects caused by the disease [8]. In clinical experience, DM patients appear to be more susceptible to facial cellulitis and deep neck infections developed from odontogenic infections [5]. This phenomenon suggests that patients of this type require a greater investment of time and resources in dental-related care than other patients to remove infections or prevent the occurrence of infections and reduce social and medical costs.

This study used the Taiwan National Health Insurance Database (NHIRD) to examine the correlation between DM and odontogenic infections in patients. The results provide suggestions for clinical treatment for and prevention of such infections in DM patients, thereby improving the quality of oral healthcare for these patients.

## Materials and methods

### Database

Since the implementation of National Health Insurance (NHI) in Taiwan in March 1995, approximately 99.9% of residents are covered under NHI [9], including foreign residents. The database of this program contains registration files and original claims data for reimbursement. Large computerized databases were derived from this system by the National Health Insurance Administration (the former Bureau of National Health Insurance, BNHI), Ministry of Health and Welfare (the former Department of Health, DOH), Taiwan. This study was exempted from full review by an institutional review board (NTUH-REC No. 201507057W) because the NHIRD consisted of deidentified secondary data released to the public for research purposes. The present study data were retrieved from 1 million randomly-sampled enrollees from the Taiwanese population. This consisted of 1 million subjects randomly selected from the entire NHI enrollee profile who represented approximately 4.5% of the Taiwanese population. The age, sex distribution, and average insured amount exhibited no significant difference between the random-sampling dataset and the overall NHIRD.

### Study design

This study analyzed NHIRD datasets of 1 million beneficiaries for 2005, of which 964,182 individuals had medical treatment records in the same year. The insurance database also recorded related factors such as age, sex, hospitalization days, season, and whether or not patients were from low-income households. A total of 23,781 newly diagnosed DM patients matched the DM diagnostic criteria and inclusion criteria set by this study. Of these, 2,271 patients met the exclusion criteria (previous record of odontogenic infections in 1997–2004, missing gender record, or aged younger than 20) and were therefore removed from the study. The remaining total of 21,510 patients meeting the criteria of this study were selected as the case group, while 43,020 non-DM patients were selected as the control group, with sex and age as the matching variables based on the 1:2 case group to control group principle. A 5-year cohort study was then conducted on the two groups.

The inclusion criteria of this study required patients to have more than 3 DM (ICD-9-CM 250) diagnoses. To determine patients with odontogenic infection, this study utilized the intra- and extra-oral incision and drainage (I&D) treatment codes (92003C, 92004C) in dental care and controlled for the comorbidity of related diseases, including hypertension (ICD-9-CM 401–405) chronic obstructive pulmonary disease (ICD-9-CM 490–496), heart disease (ICD-9-CM 410–414), congestive heart failure (ICD-9-CM 428–429), cerebrovascular accident (ICD-9-CM 430–436), and chronic renal insufficiency (ICD-9-CM 585). These diagnoses were based on those made by physicians under the standard criteria. Furthermore, this study utilized the treatment codes for addition general anesthesia intubation conducted during the same treatment (96020C-96022C or 56003C) to analyze hospitalization days and costs caused by cellulitis.

This study analyzed the correlation between urbanization and the disease in question. Past studies [10] have categorized Taiwanese urbanization into seven levels. In this study, we simplify these categories into high, medium, and low levels of urbanization. This study also defines seasons as follows: spring (March–May), summer (June–August), autumn (September–November), and winter (December–February). This study categorized patient ages as follows: 20–39, 40–59, and ≥ 60 years. Lastly, the study design was based on Strengthening the Reporting of Observational studies in Epidemiology (STROBE) guideline.

### Statistical analysis

All data were calculated with SPSS software for descriptive statistics (v22, SPSS Inc, Chicago, Illinois). Cox proportional hazards models were performed to estimate the hazard ratios in relation to DM patients with odontogenic facial cellulitis and covariates.

The Kaplan-Meier method was used to compare the incidence rate of facial cellulitis between the DM and control groups from 2005 to 2010 were evaluated through Kaplan-Meier survival analysis. A value of *p* = 0.05 was considered statistically significant. The protocols were submitted to a hospital institutional review board for review and approval was obtained (NTUH-REC No. 201507057W).

## Results

### Patients’ characteristics

**Fig 1:**
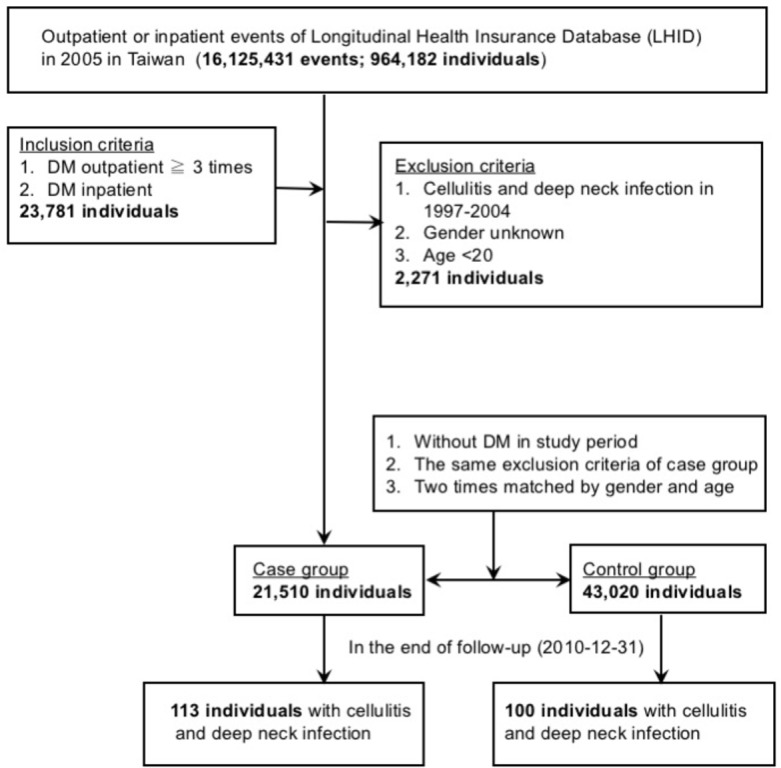
Algorithm of the study.

Of the 964,182 representative subjects included in the **Longitudinal Health Insurance Database** in 2005, 21,510 met the inclusion criteria and formed the case group. Among these patients, a total of 110 had been diagnosed with facial cellulitis stemming from odontogenic infection; these patients comprised the case group. A total of 43,020 patients matched the inclusion criteria but were not DM patients. Among these patients, a total of 100 had been diagnosed with facial cellulitis stemming from odontogenic infection; these patients comprised the control group (Fig 1). There were no significant differences between the two cohorts in demographic characteristics (Table 1).

**Table 1:** Baseline demographic status and comorbidities compared between DM and non-DM group.

DM	Total		With (Case)		Without (Control)		P
Variables	n	%	n	%	n	%	
**Total**	64,530		21,510	33.33	43,020	66.67	
**Gender**							0.999
Male	35,895	55.63	11,965	55.63	23,930	55.63	
Female	28,635	44.37	9,545	44.37	19,090	44.37	
**Age (years)** (mean±SD)	59.80±18.56		60.51±18.43		59.44±18.62		0.975
**Age (years)**							0.795
20–39	11,499	17.82	3,659	17.01	7,840	18.22	
40–59	17,791	27.57	5,728	26.63	12,063	28.04	
≧60	35,240	54.61	12,123	56.36	23,117	53.74	
**Length of days** (mean±SD)	8.40±10.90		10.45±13.13		7.37±9.43		<0.001*
**Medical costs (NT$) (mean±SD)**	54,400.26±91,068.77		64,673.05±106,591.55		49,263.87±81,734.21		<0.001*

P-value (category variable: Chi-square/Fisher exact test, continuous variable: t-test).

* A significant difference with *p* < 0.05.

### Incidence and predictors of facial cellulitis in patients with and without DM

During a mean follow-up of 5 years, the cumulative incidence of odontogenic facial cellulitis was significantly higher in the DM group compared to that of the non-DM group. As shown in Table 2, after other possible risk factors were controlled for, the risk of odontogenic facial cellulitis was higher in the DM group than in the control group (adjusted hazard ratio [HR] = 1.409, 95% confidence interval [CI]: 1.071–1.854, *p* = 0.014). After controlling for other factors, the likelihood of facial cellulitis in male patients was 1.313 times than that of female patients (adjusted HR = 1.313, 95% CI: 1.002–1.720, *p* = 0.048). However, the risk of facial cellulitis in highly urbanized areas was lower than in less-urbanized areas (adjusted HR = 0.628, 95% CI: 0.412–0.957, *p* = 0.031).

**Table 2:** Factors of cellulitis and deep neck infection in the end of follow-up by using Cox regression.

Variables	Adjusted HR	95% CI	95% CI	P
**DM**(N = 113)				
Without (Control)	Reference			
With (Case)	1.409	1.071	1.854	0.014*
**Gender**				
Male	1.313	1.002	1.720	0.048*
Female	Reference			
**Age group (years)**				
20–39	Reference			
40–59	1.125	0.748	1.888	0.262
≧60	0.833	0.542	1.198	0.338
**Catastrophic illness**				
Without	Reference			
With	1.308	0.963	1.778	0.086
**Urbanization level**				
High	0.628	0.412	0.957	0.031*
Middle	0.750	0.526	1.070	0.113
Low	Reference			

HR = hazard ratio, CI = confidence interval, Adjusted HR: Adjusted variables listed in the table

* A significant difference with *p* < 0.05.

Compared to the control group (Table 3), DM patients displayed a significantly higher risk of odontogenic infection (29.37 and 22.63 per 10^4^ person-y, respectively). The risk of odontogenic infection in the DM group overall was significantly greater than that of the female patients in the control group (adjusted HR = 1.845, 95% CI: 1.173–2.901, *p* = 0.008). Based on categorizations of disease severity, the DM group was significantly more likely to require general anesthesia for invasive debridement surgeries than the control group (adjusted HR = 2.025, 95% CI: 1.207–3.398, *p* = 0.008). Similarly, the hospitalization days and medical costs were correspondingly higher (adjusted HR = 1.409, 95% CI: 1.071–1.854, *p* = 0.014).

**Table 3:** Factors of cellulitis and deep neck infection in the end of follow-up stratified by variables listed in the table by using Cox regression.

DM	With (Case)		Without (Control)		Adjusted HR (95%CI) ^b^	P
Variables	Event	Rate ^a^	Event	Rate ^a^		
**Total**	123	29.37	104	22.63	1.409 (1.071–1.854)	0.014*
**Gender**						
Male	69	29.93	71	28.17	1.179 (0.831–1.674)	0.356
Female	54	28.69	33	15.90	1.845 (1.173–2.901)	0.008*
**Age group (years)**						
20–39	11	24.48	16	28.49	0.945 (0.675–1.296)	0.376
40–59	38	38.44	31	29.44	1.412 (0.779–1.675)	0.465
≧60	74	26.91	57	19.12	1.577 (0.976–1.945)	0.654
**Surgery**						
Without	86	28.00	76	26.28	1.182 (0.855–1.633)	0.311
With	37	33.15	28	16.44	2.025 (1.207–3.398)	0.008*
**Length of days**	123	29.37	104	22.63	1.409 (1.071–1.854)	0.014*
**Medical costs (NT$)**	123	29.37	104	22.63	1.409 (1.071–1.854)	0.014*

PYs = Person-years; Adjusted HR = Adjusted Hazard (* A significant difference with *p* < 0.05)

ratio: Adjusted for all the variables above; CI = confidence interval

^a^ Per 10^4^ person-years, derived from independent student’s test

^b^ Derived from Cox regression analysis

In less-urbanized areas (Table 4), facial cellulitis caused by odontogenic infections was also significantly more common in the DM group than in the control group (adjusted HR = 1.723, 95% CI: 1.011–2.939, *p* = 0.046). Overall, the likelihood of facial cellulitis caused by odontogenic infection was lower in autumn than in other seasons (adjusted HR = 0.572, 95% CI: 0.397–3.980, *p* = 0.010). Among patients with comorbidities, the risk of odontogenic infection was the same in the DM group as in the control group (adjusted HR = 1.308, 95% CI: 0.963–1.778, *p* = 0.086). (Table 4). Furthermore, the risk of comorbidity was higher in the DM group than in the control group (Table 5).

**Table 4:** Factors of cellulitis and deep neck infection in the end of follow-up stratified by variables listed in the table by using Cox regression.

DM	With (Case)		Without (Control)		Adjusted HR (95%CI) ^b^	P
Variables	Event	Rate ^a^	Event	Rate ^a^		
**Total**	123	29.37	104	22.63	1.409 (1.071–1.854)	0.014*
**Season**						
Spring (April-May)	28	32.33	32	32.05	1.082 (0.636–1.839)	0.772
Summer (June-August)	30	29.10	29	24.68	1.183 (0.694–2.017)	0.537
Autumn (September-November)	40	29.90	19	13.52	2.418 (1.362–4.293)	0.003*
Winter (December-Feburary)	25	26.22	24	23.60	1.284 (0.707–2.332)	0.412
**Urbanization level**						
High	34	30.19	28	20.19	1.468 (0.866–2.488)	0.154
Middle	49	26.50	53	25.18	1.161 (0.771–1.748)	0.475
Low	40	32.98	23	20.84	1.723 (1.011–2.939)	0.046*
**Surgery**						
Without	86	28.00	76	26.28	1.182 (0.855–1.633)	0.311
With	37	33.15	28	16.44	2.025 (1.207–3.398)	0.008*

PYs = Person-years; Adjusted HR = Adjusted Hazard

ratio: Adjusted for all the variables above; CI = confidence interval

^a^ Per 10^4^ person-years, derived from independent student’s test

^b^ Derived from Cox regression analysis

* A significant difference with *p* < 0.05

**Table 5:** Factors of cellulitis and deep neck infection in the end of follow-up stratified by variables listed in the table by using Cox regression.

DM	With (Case)		Without (Control)		Adjusted HR (95%CI) ^b^	P
Variables	Event	Rate ^a^	Event	Rate ^a^		
**Catastrophic illness**						
Without	90	28.32	65	19.03	1.493 (1.072–2.080)	0.018*
With	33	32.66	39	33.07	1.073 (0.644–1.789)	0.787
**HTN**						
Without	81	28.44	88	24.01	1.336 (0.974–1.833)	0.072
With	42	31.35	16	17.20	1.753 (0.968–3.176)	0.064
**COPD**						
Without	114	30.88	101	23.44	1.406 (1.062–1.863)	0.017*
With	9	18.14	3	10.46	1.407 (0.355–5.582)	0.627
**CRI**						
Without	116	28.85	102	22.61	1.400 (1.059–1.852)	0.018*
With	7	41.71	2	23.44	1.320 (0.234–7.452)	0.753
**IHD**						
Without	109	30.07	103	23.79	1.330 (1.003–1.763)	0.047*
With	14	24.85	1	3.76	5.832 (0.749–45.408)	0.092
**CHF**						
Without	112	28.98	104	23.69	1.335 (1.008–1.766)	0.044*
With	11	33.97	0	0.00	-	0.947
**CVA**						
Without	119	29.99	104	23.29	1.384 (1.049–1.825)	0.021*
With	4	18.17	0	0.00	-	0.749

PYs = Person-years; Adjusted HR = Adjusted Hazard

ratio: Adjusted for all the variables above;

CI = confidence interval

HTN = Hypertension: ICD-9-CM 401–405;

COPD = Chronic obstructive pulmonary disease: ICD-9-CM 490–496;

CRI = Chronic renal insufficiency: ICD-9-CM 585

IHD = Ischemci heart disease: ICD-9-CM 410–414;

CHF = Congestive heart failure: ICD-9-CM 428–429;

CVA = Cerebrovascular accident: ICD-9-CM 430–436

^a^ Per 10^4^ person-years, derived from independent student’s test

^b^ Derived from Cox regression analysis

* A significant difference with *p* < 0.05

**Fig 2:**
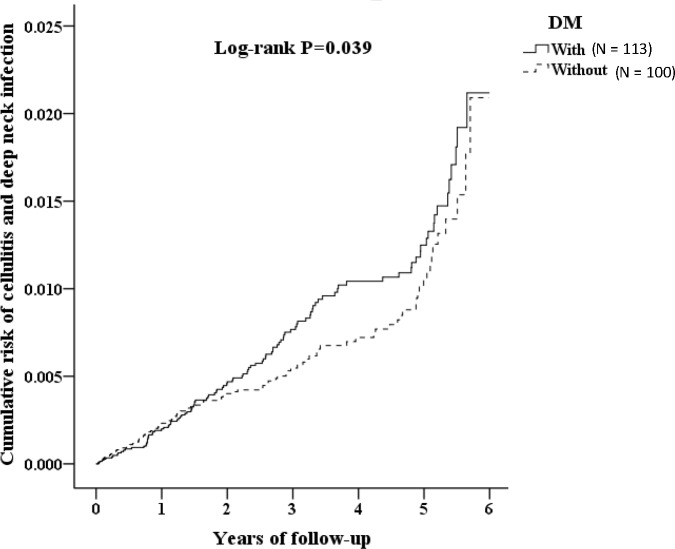
Kaplan-Meier for cumulative incidence of facial cellulitis from odontogenic infection in patients by log-rank test.

We used Kaplan-Meier survival analysis to assess the cumulative incidence of odontogenic infection. The differences in cumulative incidence among the groups with or without DM were significantly different (log-rank test; *p* = 0.039; Fig 2).

## Discussion

The development of modern medicine, infectious disease control, and the prevalence of obesity in modern society has led to the emergence of DM as one of the most critical public health issues in the 21st century. In Taiwan, the incidence and prevalence of DM is rapidly rising. Statistical estimates indicate a total of 190 million DM patients worldwide, accounting for 9% of the global adult population. In 2012, approximately 1.5 million deaths were directly caused by DM [11]. WHO estimates that there will be 330 million DM patients in 2025, and by 2030, DM will become the seventh leading cause of death worldwide [12]. In Taiwan, the current prevalence of DM is approximately 4% of the population [13]. The broad impact of DM is caused not only by the abnormal increase of blood glucose associated with the disease, but also changes in lipid, protein and carbohydrate metabolism [14] and the subsequent development of diseases such as coronary and lower limb vascular disease and nephropathy [15]. In addition, clinical evidence indicates that poorly controlled DM increases wound-healing time and the likelihood of bacterial and fungal infections. However, the relationship between facial cellulitis and deep neck infections caused by odontogenic infections and DM, as well as other factors impacting related comorbidity have not yet been closely examined.

The present cohort contained all DM patients from multiple hospital units across the country. Universal Taiwan NHI coverage, supported by the government, provides every patient with unrestricted access to medical services from any healthcare provider. All DM patients seeking treatment for facial cellulitis were included in this study, with the exception of those treated abroad or those without NHI coverage. Thus, the uniquely comprehensive feature of the database enhances the accuracy and representation of the estimation of disease incidences. Past studies have indicated that DM patients are more likely than non-DM patients to develop deep neck infections [6,16–17]. However, because of the complex and possibly interfering comorbidities associated with DM, although the correlation between DM and deep neck infection has been proposed in the past, this hypothesis was highly arguable. However, this study used data from the NHIRD to observe the incidence of facial cellulitis among DM patients. Of 21,510 patients diagnosed with DM, 113 developed facial cellulitis as a result of odontogenic infection. This nationwide retrospective cohort study demonstrates that after controlling for all other systemic diseases, the possibility of facial cellulitis developing from odontogenic causes among DM patients is 1.409 times than that of normal groups. The cases covered in this study included not only those involving deep neck space infections, but also other comparatively milder cases involving facial spaces, such as the buccal and canine spaces. Because the oral cavity itself is an open space, odontogenic infections can easily cause fistulas or swelling around wisdom teeth through root infections in clinical practice, thereby causing further cervical lymphadenitis and abscess formation. Odontogenic infections generally spread from the mandible or maxilla to sublingual spaces, submandibular spaces, or masticatory spaces [18] before extending to the parapharyngeal space. To summarize, the discovery that DM patients are 1.409 times more likely than non-DM patients to develop facial cellulitis from odontogenic infections appears to be clinically significant.

As the clinical significance of the association between DM and facial cellulitis has not yet been conclusively determined, in this study, we conducted a complete and well-executed Kaplan-Meier analysis. This study found that odontogenic facial cellulitis exhibits a significantly higher incidence two years after DM diagnosis. In previous studies, comorbidity has been shown to occur frequently among patients with diabetes [19]. The relation between hypertension and youths with type 2 DM has been established and the prevalence of hypertension was over 30 percent with mean duration of 8 months [20]. On the other hand, patients with diabetes have an alteration in collagen metabolism [21], oral vascularization [22], neutrophil function and reduced phagocytic capacity and chemotaxis [23]. In addition, one study revealed elevations in inflammatory markers with high sensitive C-reactive protein, homocysteine, and plasminogen activator inhibitor-1, and increased in serum levels over 36 months of follow-up [24]. These immunological dysfunctions and provoked inflammation may aggravate previous oral pre-existing problems as dental caries, periodontitis/pericoronitis, oral ulcer or poor oral hygiene to possibly render different statuses of acute or chronic infection, and may give us a reasonable explanation of the association between the higher incidences of odontogenic facial cellulitis and 2 years after DM diagnosis. (Fig 2).

Furthermore, this study also found that female patients diagnosed with DM are more likely to develop odontogenic cellulitis compared to non-DM female patients. Because DM decreases patient immune-system function, DM patients are a high-risk group for opportunistic infections. Past studies on gender groups [25] have indicated that gender has no significant correlation with DM and does not have an effect on subsequent pathological changes. However, past DM studies have also indicated that female patients experience a greater degree of difficulty in controlling cholesterol, blood pressure, and body weight during both menopausal periods [26] and gestational [27]and because of the effects of hormones [28], thereby complicating the management of cardiovascular health and DM. These findings on DM pathological changes are also consistent with the results of this study. Furthermore, this study found that the treatment of DM patients is more likely to require general anesthesia and longer hospitalization, thereby generating higher overall medical costs than those incurred by non-DM patients. These results show that DM itself causes a more rapid progression of cellulitis and more serious infections [29]. Furthermore, because inflammation causes insulin resistance and increases blood glucose levels [30], the increased difficulty of blood glucose management along with impaired wound healing caused by DM [31,32] are both possible factors increasing hospitalization stays and medical costs.

According to statistics published by the American Diabetes Association in 2013, spending in 2012 on DM reached US$245 billion, with US$176 billion accounted for by direct medical costs and US$69 billion accounted for by lost patient productivity. In addition, the annual medical costs spent on each diagnosed DM patient total US$13,700 on average, with approximately US$7,900 spent on medical care directly related to DM. On average, medical costs incurred by patients diagnosed with DM are approximately 2.3 times higher than those incurred by patients without DM. In addition to the financial burden caused by the considerable medical costs, DM may also lead to more serious health problems including cardiovascular and renal disease, poor blood circulation, amputation, problems with vision, blindness, and premature death; these health impacts also affect patient productivity, thus producing a vicious cycle [33]. Although Taiwan NHI coverage reduces the economic burden on patients, many studies indicate that disadvantaged socioeconomic groups exhibit poorer patient self-care [34], resulting in greater difficulty in controlling DM symptoms [35]. Thus, we can surmise that the risk of odontogenic facial cellulitis is higher for these patients. The above factors may be among the causes of the higher incidence of odontogenic facial cellulitis among DM patients.

In addition, this study found that the incidence of odontogenic facial cellulitis in the DM group was the lowest during autumn. These results coincide with our clinical experience, although no past studies have directly offered this conclusions. However, past studies [36–38] have indicated that cold weather may detrimentally impact the glycated hemoglobin value of DM patients because seasonal changes are associated with greater difficulty in managing DM. As such, a higher incidence of problems associated with dental infections is plausible. By contrast, glycemic control is relatively easy during the comparatively warm autumn season. Furthermore, DM patients may exhibit a greater degree of willingness to seek treatment earlier because of the comfortable weather.

## Limitation

Because of the potential error produced by inconsistent NHI treatment codes and subsequent inconsistency in diagnoses, the analyses presented in this study did not involve the treatment code for facial cellulitis, but used dental procedure codes (intra-oral and extra-oral, I & D) instead. Because Taiwan uses an insurance reimbursement system that divides different medical departments, only dental and oral maxillofacial surgeons submit dental operation-related data under the corresponding NHI codes. Furthermore, based on the literature review conducted in this study, approximately 90% of head and neck infections originate from dental disease [3]. As a result, we believed that examining operation codes would provide a greater degree of accuracy in filtering and identifying patients who have received treatment for odontogenic infections. In this study, the cases covered in this study included not only those involving deep neck space infections, but also other relatively milder cases involving facial spaces, such as the buccal and canine spaces. However, the ambiguity in the definition of dental operation codes rendered the severity of cellulitis difficult to be clearly distinguished. This study therefore cannot definitively demonstrate the severity of cellulitis.

It is of interests to note that data in the National Health Insurance Research Database (NHIRD) is scrambled before being sent to the NHRI for database construction and is further scrambled before being released to each researcher. It is impossible to query the data alone to identify individuals at any level using this database. Therefore, when odontogenic infectious diseases occurred or the effect of disease duration could not be presented by this study. However, DM increases the susceptibility to infections due to an altered immune response [39–41] and over 50% patients suffering from necrotizing fasciitis have been *associated* with DM [42]. Another study showed persistent poor glycemic control has been associated with a greater incidence and progression of gingivitis and periodontitis [41,43,44]. Consequently, poor glycemic control over time has been linked to the development periodontal putative pathogens accumulation [45], it is predictable to increase the infection severity, length of days and medical costs.”

Another limitation of this study is the lack of information in the NHI database on the degree of glycemic control in DM patients. Although DM patients were defined by this study as those diagnosed twice consecutively with DM, the NHI database does not provide any information on the circumstances of patient glycemic control. As such, this study cannot confirm whether all DM patients with different circumstances of glycemic control are more likely to develop facial cellulitis than healthy individuals, or only poorly-controlled DM patients are more likely to develop the condition. However, because poorly-controlled DM is widely considered to affect wound healing and increase the likelihood of infection [46], this study proposes that the primary risk group is poorly-controlled DM patients.

## Conclusion

The study provides a new viewpoint on the association of odontogenic infection with DM. A nationwide survey of the incidence of facial cellulitis caused by odontogenic infection in patients with DM over a span of more than a decade offers realistic statistics of the prevalence of this condition, and might be helpful in understanding the natural history of facial cellulitis in DM patients. In this large cohort, the relationship between facial cellulitis and DM patients was confirmed, with the condition most likely to occur 2 years after initial DM diagnosis and with a risk 1.409 times that of the normal population. Odontogenic cellulitis has also been observed to occur less often during autumn and more often in disadvantaged socioeconomic groups. Furthermore, female DM patients are more likely to develop odontogenic facial cellulitis. Although developments in medicine have led to a decline in the incidence of deep neck infections, this condition remains a primary cause of head and neck morbidity and mortality. The conclusions of this study can form the basis of guidelines for preventative education and treatment. Early oral prevention and health education for high-risk patients, as well as surgical intervention and antibiotic usage in early-stage odontogenic infections, can prevent disease progression, accelerate patient recovery, and reduce the use and waste of medical resources.
